# A novel microfluidic approach to quantify pore-scale mineral dissolution in porous media

**DOI:** 10.1038/s41598-025-90429-x

**Published:** 2025-02-21

**Authors:** Rafid Musabbir Rahman, Elliott Niemur, Gianluca Blois, Farzan Kazemifar, Myeongsub Kim, Yaofa Li

**Affiliations:** 1 Department of Mechanical & Industrial Engineering, Montana State University, Bozeman, MT 59717 USA; 2 Department of Mechanical Engineering, University of Idaho, Boise, ID 83702 USA; 3 Department of Mechanical Engineering, San Jose State University, San Jose, CA 95192 USA; 4 Department of Ocean and Mechanical Engineering, Florida Atlantic University, Boca Raton, FL 33431 USA; 5 Department of Mechanical Engineering, University of California, Riverside, Riverside, CA 92521 USA

**Keywords:** Mineral dissolution, Microfluidics, Porous media, Calcite, Pore scale, Multiphase flow, Geochemistry, Hydrology, Climate-change mitigation

## Abstract

Mineral dissolution in porous media coupled with single- and/or multi-phase flows is pervasive in natural and engineering systems. Dissolution modifies the physical, hydrological, and geochemical properties of the solid matrix, resulting in a complex coupling between local dissolution rate and pore-scale flow. The work reports a microfluidic approach that includes 2D reactive porous media and advanced pore flow diagnostics for the study of pore-scale dissolution in porous media with unprecedented details. The 2D microfluidic porous media, called micromodels, were fabricated in calcite by combining photolithography and wet etching, which not only offers precise control over the structural and chemical properties, but also facilitate unobstructed optical access to the pore flow, significantly improving over existing methods. We believe the work represents the first of its kind as it for the first time directly applies photolithography to calcite samples and demonstrates the use of particle image velocimetry to investigate chemical reactions in porous media. The preliminary results have revealed the crucial roles of local concentration gradients in mineral dissolution and call for reconsideration of many assumptions used in the current modeling tools, which paves the way for renewed fundamental understanding of reactive transport and improved modeling tools with better accuracy.

## Introduction

Reactive dissolution of minerals in porous media is pervasive in natural and engineering flow systems^1–8^. These processes play a defining role in a broad range of applications including: (i) carbon capture and sequestration (CCS)^1–4^; (ii) soil formation and contaminant transport^5,6^; (iii) development of underground cave systems^7^; and (iv) global carbon and nutrient cycling^8^. For instance, geologic carbon sequestration is widely considered as a viable technology to help reduce carbon emissions to the atmosphere. However, injection of CO$_2$ into geologic formations promotes erosion via dissolution of rock minerals, potentially compromising the integrity of the reservoir rocks cap by creating leakage pathways that threaten the safety and stability of CO$_2$ containment^1,2^. In agriculture, mineral dissolution supplies nutrients to soil^8^, but at the same time it can also cause the release of heavy metals, which causes soil contamination and complicates potential cleanup processes^5,6^.

With the advances of theoretical, experimental and numerical studies, especially the use of kinetic Monte Carlo models and the density function theory (DFT), our understanding of the kinetics, thermodynamic, and chemical aspects of dissolution processes in hydrologically static scenarios has significantly improved^9,10^. However, the coupled fluid mechanics involved in a flow system remain elusive, particularly in geometrically complex domains such as a porous matrix. The reason for this is that the dissolution of rock minerals simultaneously modifies the physical properties and chemical composition of the solid matrix, which in turn alters the chemistry and transport in the aqueous flow^11^. These two processes are inherently coupled thus controlling the evolution of the process both at the microscopic and at the field scale^12^. Moreover, naturally occurring porous media are often of high heterogeneity, both structurally and chemically, which span enormous ranges of scales both spatially and temporally. Processes at fine (pore) scales are known to meaningfully impact dissolution at much larger (reservoir) scales^13^. In fact, one of the most quoted challenges in the literature is that dissolution rates in porous media measured in the lab are typically orders of magnitude higher than those observed in the field, often referred to as the “lab-field discrepancy”^14–20^. The mismatch not only poses strong challenges in developing accurate predictive models based on the rate laws developed in laboratory, but also highlights a lack of fundamental understanding of mineral dissolution in porous media, especially at the pore scale, which hinders our ability to successfully model, control and optimize many of those aforementioned processes.

In the past few decades, extensive studies have been devoted to reconciling this “lab-field discrepancy” using experimental, numerical and theoretical approaches^15,16,18,20^. While the exact cause(s) of the enormous discrepancy remains the object of scientific debate, it has been conjectured that a fundamental component of real reactive transport phenomena is being overlooked at best and/or completely missed, due to the way current studies are conducted. For example, it is well accepted that laboratory measurements based on well-mixed systems (i.e., with no or minimal concentration gradient) do not appropriately represent the real scenario, which are often subject to transport and concentration variability^14^. Several studies have proposed that the lab-field discrepancy is primarily due to concentration gradients resulting from spatial variability in dissolution and incomplete mixing within individual pores^15,16,18,20^. Such variability produces complex local flow patterns such as preferential flow paths and mixing regions that are not observable through traditional methods. The kinetics of the reaction strictly depend on such flow patterns, rendering pore flow quantification a critical component in solving this problem.

In that sense, microfluidic model porous media (i.e., micromodels) combined with light microscopy has emerged as a powerful tool to produce such measurements^21–28^, for its simplicity, vast availability, excellent flexibility and high temporal and spatial resolving power compared to other approaches like microscopic computed tomography (micro-CT)^29,30^ and nuclear magnetic resonance (NMR)^31,32^. Two-dimensional (2D) porous micromodels feature optically accessible interconnected porous networks that allow for direct imaging of pore flows when combined with advanced flow diagnostic techniques such as microscopic particle image velocimetry (micro-PIV)^33–35^. This approach has enabled the discovery and quantitative analysis of numerous complex flow mechanisms in porous media, including transient events associated with immiscible multi-phase interactions, such as Haines jump^36^, inertial effect^22^, fingering^23,37^ and shear-induced recirculation^38^. Traditional micromodels are typically made of chemically inert materials including glass, silicon, and polymers like polymethyl methacrylate (PMMA) and polydimethylsiloxane (PDMS). In addition to their high manufacturability, these materials are used due to their low chemical reactivity with common working fluids^39–41^. While these non-reactive micromodels have been serving as excellent platforms to investigate physical processes and mineral precipitation^11,42,43^, they are not suitable or not ideal for studies where erosive reactions of the solid matrix are expected. For such cases, it is critical to fabricate the micromodels using materials that are chemically reactive as well as geologically representative of the subsurface environment.

Several previous studies have explored options based on either a “top-down” or a “bottom-up” approach^44^. Top-down strategies often employ traditional micro-milling and etching combined with laser cutting and patterning for better precision^45^, whereas bottom-up strategies are primarily inspired by biomineralization^44^. The work by Song et al.^45^ is among the first studies to have successfully fabricated a calcite-based micromodel using laser cutting and wet etching. The process started with thin-sectioning a large block of natural calcite crystal to create a 3 mm thick calcite wafer, following which a porous pattern was generated with the aid of a layer of beeswax and a laser cutter. The micromodel was completed by wet etching in hydrochloric acid, drilling and bonding to another piece of glass. While this approach is innovative, its precision is relatively limited, with a minimum feature size of approximately 140 $\upmu$m. Additionally, micromodels created using this method have inlet, outlet, bounding wall, and the porous section all made in calcite, inevitably causing reactions to take place everywhere: both within and outside the porous section; and in the third dimension normal to the bottom wall of the microchannel, which may interfere with the reaction and flow in the region of interest. Soulaine et al.^4^ and Rembert et al.^46^ adopted the similar idea of using a thin calcite slice that is pre-machined, and the thin calcite of a pre-defined shape was then directly embedded in a straight PDMS microchannel. Although the PDMS channel helped to eliminate the unwanted outer reaction, the method provided limited control over the geometry of the post, and the entire micromodel consisted of only one calcite post, hardly justified as a porous medium. To take one step further, Singh et al.^47^ embedded a real rock slice of 500 $\upmu$m thick, into a PDMS channel. Thanks to the use of real rock slices, this micromodel is faithfully representative of both real geometry and geochemistry thus being ideal when quantifying global quantities such as reaction rates and pressure drops. However, the use of such natural material makes each micromodel different and unique in terms of physical, geological and chemical properties, hindering necessary repeatability test at the pore scale as well as the possibility of parametric studies of pore flow using control variables. Moreover, the opaqueness of the rock sample renders the optical-based measurement challenging and sub-optimal.

**Fig. 1:**
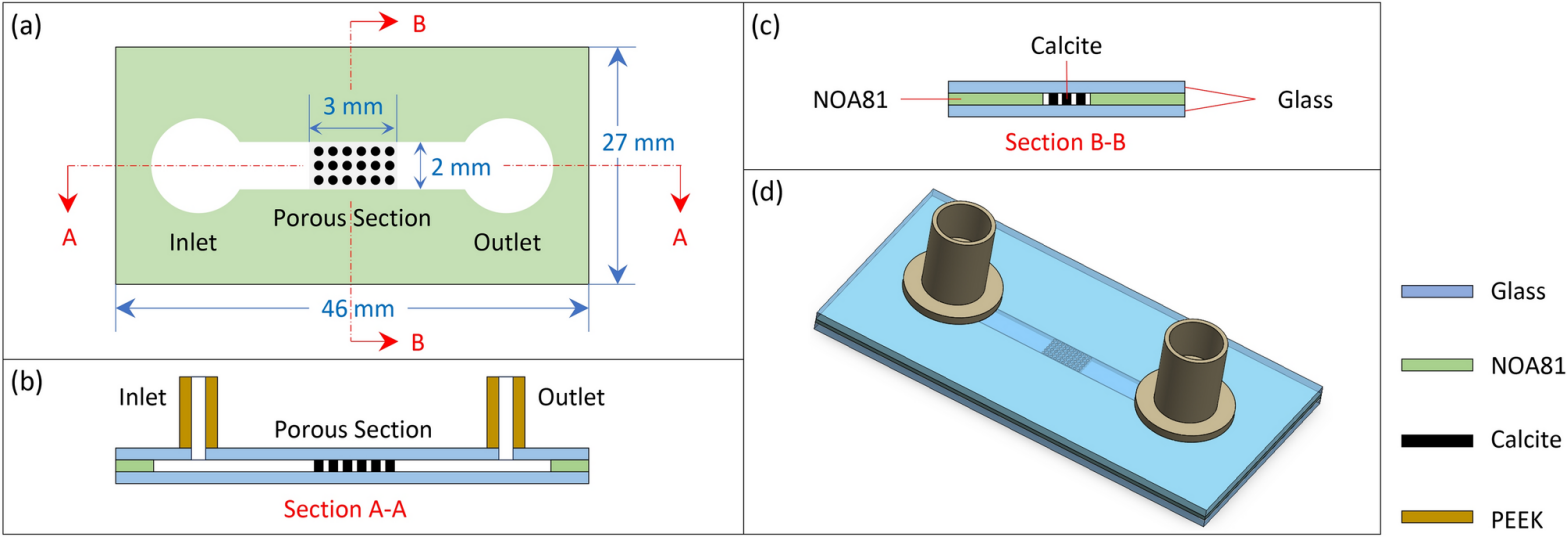
Schematic diagrams illustrating the design of the micromodel: (**a**) top, (**b**) side and (**c**) cross sectional views. The micromodel consists of two layers of glass (blue) with a thin calcite (black) layer sandwiched in between and surrounded by the UV glue layer (NOA81 herein as shown in green). In this particular design, the porous section is formed by regularly arranged circular posts. Panel (**d**) shows a perspective view of the entire device with all three layers assembled and two PEEK nanoports attached for fluid delivery. Drawings are not to scale.

Bottom-up approaches have attempted to grow reactive minerals (e.g., CaCO$_3$) in-situ within a traditional non-reactive micromodel. Lee et al.^44^ developed a technique to selectively grow CaCO$_3$ in a glass microchannel to form a porous section. The method is enabled by a UV-curable precursor solution, which creates preferential sites for CaCO$_3$ growth. Upon delicate controls of the rinsing and precipitating flows, CaCO$_3$ posts were successfully grown with pre-defined geometry. This approach, while novel and precise, requires sophisticated control of various parameters and is of low throughput. Poonoosamy et al. fabricated PDMS micromodels using standard soft lithography and mechanically injected celestine crystals to create 3D reactive porous micromodels^48,49^. Wang et al.^50^, Song et al.^51^, and Alzahid et al.^52^ took a similar approach by fabricating a standard micromodel in glass, silicon and PDMS, respectively and grew a thin layer or grains of CaCO$_3$ crystals within. The approach is relatively straightforward, but the major drawback is that the original surfaces (i.e., glass, silicon or PDMS) of the micromodels are either not fully covered with CaCO$_3$, or only by a thin layer of CaCO$_3$ of *O*(1 $\upmu$m) thick, making them unsuitable for studies of dissolution rate or prolonged fluid-mineral interactions. Moreover, all the aforementioned bottom-up approaches based on CaCO$_3$ growth are susceptible to a certain level of uncontrolled processes, i.e., nucleation sites and crystal structures, raising concerns over experimental reproducibility.

To further improve our ability to study mineral dissolution at the pore scale, we herein introduce a new method to create calcite-based micromodels combining traditional rock sample preparation techniques (i.e., thin sectioning and grinding) with semiconductor technologies such as photolithography, wet etching and adhesive bonding. Inspired by many previous approaches, this new method effectively bears most previous advantages such as high precision and easiness of fabrication while overcoming most disadvantages, such as low precision and uncontrolled/unwanted reactions. These new micromodels not only offer precise control over the structures and chemical properties, but also facilitate previously unattainable unobstructed and unaberrated optical access to the pore flow around calcite-based micropillars. Moreover, using the micro-PIV technique and epi-fluorescence microscopy we gained valuable insights into mineral dissolution with unprecedented details and spatial resolution. While standard photolithography is almost always performed on glass or silicon wafers, in this work we demonstrate for the first time that the use of photolithography with calcite is appropriate.

## Methods

### Micromodel design

**Fig. 2:**
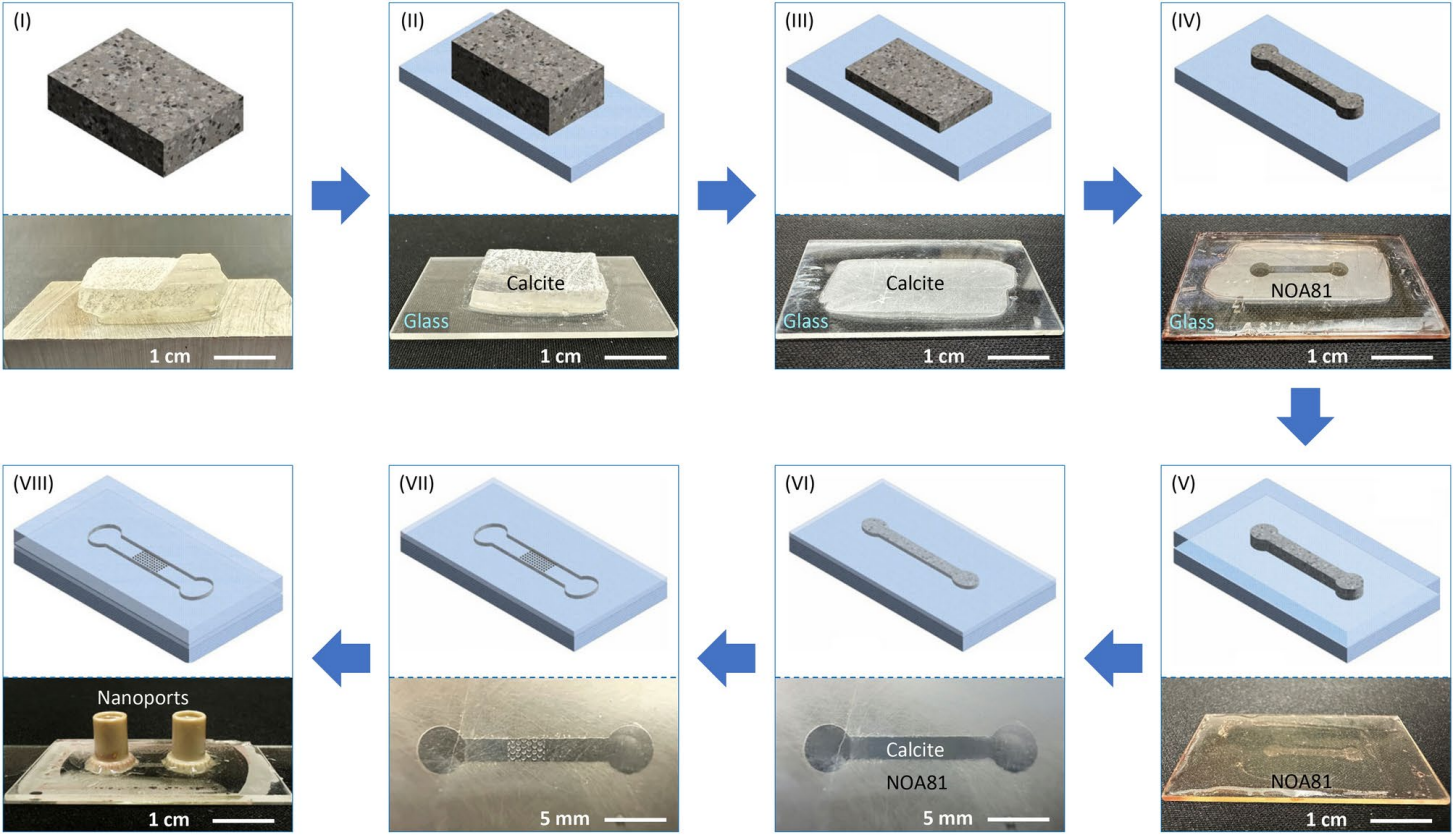
3D sketches illustrating the major steps required to fabricate a calcite-based micromodel. The top half of each panel shows the cartoon of the particular step, whereas the bottom half shows the actual outcome of the step. (**I**) A bulk calcite crystal (VWR, Iceland Spar) is sectioned and polished at the top surface. (**II**) The polished surface is attached to a glass slide using UV glue, and (**III**) grounded to 100–120 $\upmu$m. (**IV**) Employing standard photolithography and wet etching in diluted HCl solution, the calcite layer is shaped into a microchannel. (**V**) A layer of UV glue is uniformly spread over the etched calcite and cured. (**VI**) The cured UV glue along with the covered calcite is ground to approximately 30 $\upmu$m. At this point, the entire microchannel is filled with calcite which is surrounded by UV glue. (**VII**) Again employing photolithography and wet etching, the calcite porous pattern is formed whereas the rest part of the calcite is etch out, exposing the microchannel. (**VIII**) Two through holes are drilled into the glass slide for fluid delivery, and the sample is then adhesively bonded to another glass wafer to form a closed microchannel. Finally, the nanoports were attached to complete the fabrication process.

The design of the micromodel is primarily guided by three considerations: (i) the geometry of microchannel has to be precisely controlled and conveniently customized when necessary; (ii) the porous section needs to be reactive while the boundaries ought to be non-reactive to warrant controlled flow in the porous section; (iii) the device ought to provide optical access to enable epi-fluorescent microscopy and micro-PIV. Based on these requirements, the design is schematically illustrated in Fig. 1. The overall size of the device is $46\times 27\times 2$ $(L\times W\times H)$ mm$^3$, matching the shape and size of a standard glass slide (Ted Pella, Inc. Product NO:260600), which is used as the base of the device.

**Fig. 3:**
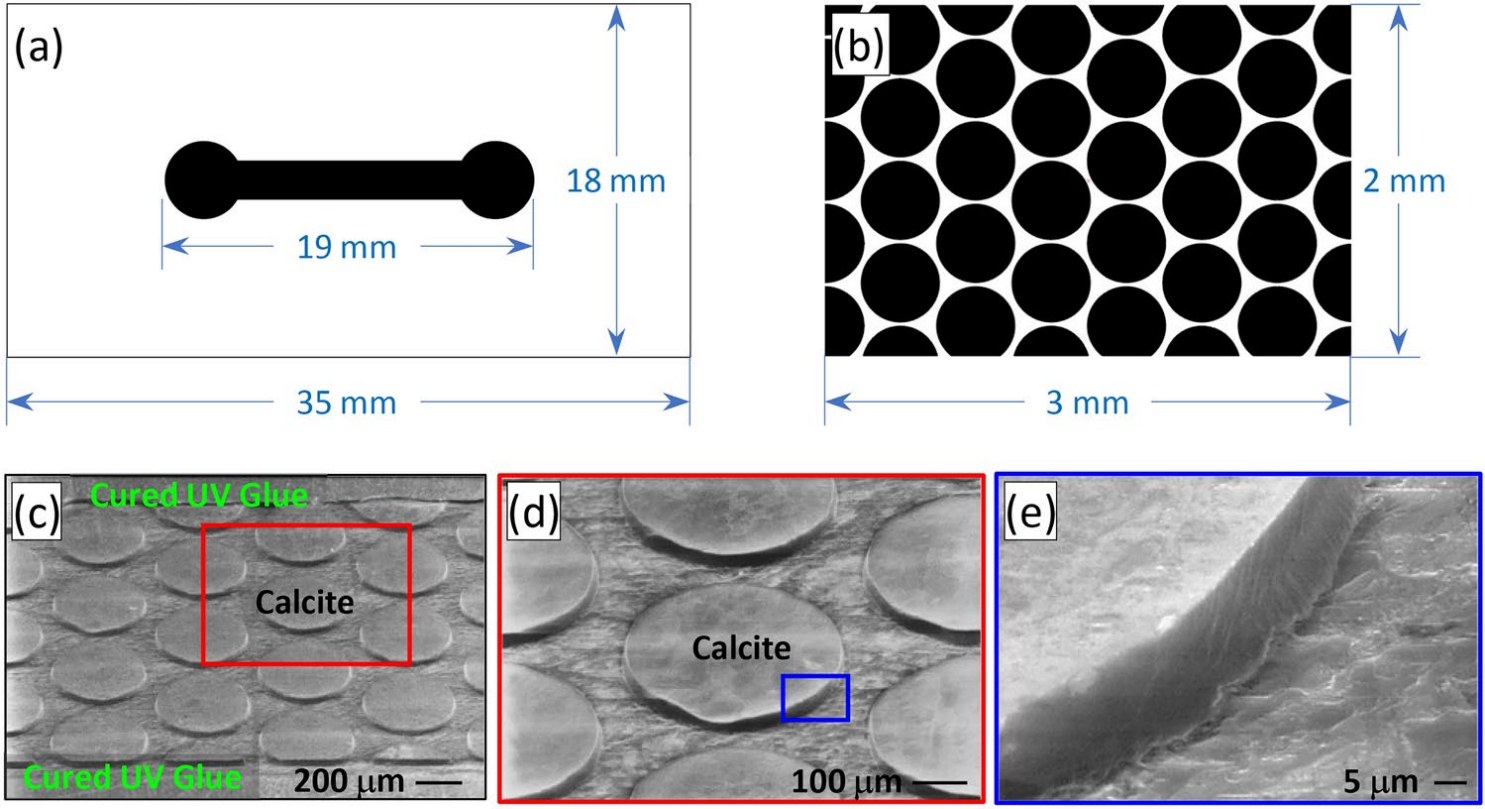
The two photomasks used in the photolithograpy processes (a and b, i.e., designed structure) and SEM photos of the calcite posts after wet etching (**c–e**, i.e., achieved structure). Masks I (**a**) and II (**b**) are used to form the main microchannel and the porous section, respectively. The photo shown in panel (**d**) is a blown-up view of the region enclosed by the red rectangle in panel (**c**), illustrating smooth top surfaces and clean edges of the calcite posts; panel (**e**) shows a further blown-up view of the region enclosed by the blue rectangle in panel (**d**), demonstrating the very smooth lateral surfaces of the etched pillars with sub-micrometer roughness.

As shown by the top view in Fig. 1a, the micromodel consists of a main channel, an inlet, an outlet and a porous section. The main channel is 19 mm long, 2 mm wide and 30 $\upmu$m deep. While the top and bottom of the main channel are bounded by two glass slides (see panels b and c), the in-plane structure is formed in UV glue (Thorlabs NOA81). The porous section of $3\times 2$ mm$^2$ is fabricated in calcite, which is reactive to acidic flows such as hydrochloric acid (HCl) and carbonated water, and facilitates mineral dissolution experiments. Although the porous section can be made to virtually take any shape or structure, in this particular demonstration, the porous section is formed with regularly arranged circular posts with a porosity of approximately 20.6% (c.f., Fig. 3)b, mimicking the porosity of a real porous structure such as calcite formation and soil^53^. The calcite porous section is surrounded by a layer of UV glue with the same thickness that confines lateral flow, whereas the calcite porous section and UV glue layer are both sandwiched between the two glass slides on top and bottom to confine vertical flow. This innovative design ensures that the porous section is reactive, while the bounding channel is non-reactive. The design is crucial to producing controlled 2D flow and limiting reactions in the porous section while eliminating unwanted interference from the upstream, the downstream or in the third dimension, which was not possible in previous designs^45,47^.

It is worth noting that the UV glue is chosen here for its high versatility to mold various shapes. The particular UV glue used here is originally in a liquid form with a dynamic viscosity of 300 mPa s and it cures into solid within seconds under the exposure of UV light, making them well suited to molding various shapes and providing adhesive bonding between different layers. More importantly the wettability of the UV glue can be altered by exposing it to deep UV light, offering a good potential to study the effect of wettability^54^. While exploring the effect of wettability is beyond the scope of the current study, it will certainly be the object of our future work employing the method outlined herein. Additionally, the transparent glass slides allow for clear and unobstructed optical access to the porous structure, facilitating direct observations and analysis of the flow and dissolution processes in real time using optical microscopy and micro-PIV. Finally, the inlet and outlet serve as the entry and exit points for introducing and removing working fluids within the micromodel through the two nanoports.

### Micromodel fabrication

While this micromodel design is novel, it poses two major challenges to fabrication. First, the microchannel is to be formed in UV glue with a pre-defined geometry and thickness of 30 $\upmu$m which cannot be achieved with traditional machining processes nor more recent 3D printing due to limitations on material selection and spatial resolution. Second, the calcite posts, hereinafter, referred to as grains, need to be planted to the microchannel precisely to prescribed locations to form a pre-defined pattern. These challenges are overcome herein by creatively combining traditional rock machining procedures with microfabrication techniques such as photolithograpy and wet etching as detailed below. The fabrication procedures are illustrated in Fig. 2 consisting of eight major steps. First, a bulk calcite crystal (VWR, Iceland Spar, 470025-522) of approximately $30\times 20\times 5$ mm$^3$ was ground and polished at the top surface using a grinder (Allied High Tech 5-2600) combined with diamond discs (Allied High Tech 50-80800) and the alcohol-based diamond slurries (Allied High Tech 90-3AB6-G) (Fig. 2I). These Iceland Spars are commercially available and were used as received. Then the polished calcite surface was cleaned with acetone and attached to a glass slide (Ted Pella, Inc. Product NO:260600) using the UV glue (Thorlabs, NOA81), which was cured with a UV lamp for 30 minutes while the sample was securely clamped. Then the clamp was removed and the sample was cured for another 30 minutes under the UV light to ensure secure bonding between the calcite layer and the glass slide (Fig. 2II). Following the bonding step, the other surface (now at the top) of the attached calcite was ground using silicon carbide papers. The grinding process started with a silicon carbide paper with a grit size of 400 and gradually refined to the grit size 800 to ensure efficiency as well as grinding quality. The targeted thickness of the calcite in this step is 100–120 $\upmu$m (Fig. 2III). Specifically, the process started with a 400 grit paper for 3 min and 45 s, following which a 600 grit paper was used for 1 min and the process was completed with a 800 grit paper for 5 min resulting in a calcite thickness of approximately 118 $\upmu$m as measured by a digimatic micrometer (Mitutoyo, 293-821-30). These polishing and grinding processes were again done on the Allied High Tech Metprep 3$^\text {TM}$ grinder/polisher with a power head (Allied High Tech 5-2600).

At this point, a uniform layer of calcite of approximately 118 $\upmu$m was successfully formed on the glass slide, which was then ready for the first photolithography process. To perform photolithography, a photomask (Mask I) was designed as shown in Fig. 3a which carries the shape of the main channel. The photomask was designed in Adobe Illustrator^®^ and printed by a third-party company (CAD/Art Services, Inc.). A 9 $\upmu$m thick layer of photoresist (MicroChemicals, AZ^®^10XT) was spin-coated on the calcite sample at a spinning speed of 2000 rpm for 45 s. Then the sample was soft baked for 6 min at 115 $^{\circ }$C, following which the sample was allowed to rest for 1 hour to thermally equilibrate with the environment. The photoresist was subsequently exposed under UV light at 3.36 mW/cm$^2$ for 106 s, and developed in a photoresist developer (MicroChemicals, AZ^®^300 MIF) for 15 min. Finally, the sample was rinsed with DI water and dried with high-purity nitrogen stream to complete the photolithography process. Then the calcite layer was wet etched in 2% hydrochloric acid (HCl) for 4 min or until the uncovered portion was all etched through. As shown in Fig. 2IV, these procedures produce a thick calcite layer of 118 $\upmu$m that is adhesively bonded to the glass slide and shaped as the microchannel. This dumbbell-shaped calcite is critical and serves for two purposes in the following steps: (i) it supplies calcite material to form the porous section; (ii) it is used as the mold to form the microchannel in UV glue in the next step.

A layer of UV glue was dispensed over the dumbbell-shaped calcite to cover the glass slide. The glue was degassed under vacuum for 30 min to remove any bubbles from the glue, following which the glue was cured for 10 min under UV light (Thorlabs, SOLIS-365C), resulting in an approximately 0.5 mm thick UV glue layer covering the calcite (Fig. 2V). The cured glue along with the covered calcite was then ground using the same Allied High Tech Metprep 3$^\text {TM}$ grinder/polisher with the power head (Allied High Tech 5-2600) to reach a thickness of about 30 $\upmu$m. For this step, silicon carbide paper of 600 grit size was used for 6 min and 50 seconds, eventually exposing the dumbbell-shaped calcite at the top surface, which is surrounded by the cured UV glue (Fig. 2VI). The polished sample was then taken to perform the second photolithography process with a second mask (Mask II) as shown in Fig. 3b to form the porous structures on the calcite. The materials and protocols are largely the same as the first photolithograpy process with only one difference that the UV exposure of the photoresist was done with the aid of a customized mask aligner, to ensure the porous pattern is well-aligned with the calcite. Following a sequential processes of UV exposing, developing, and cleaning, the sample was etched again by immersing it in 0.5% HCl solution for 10 min. After etching, the regions that were covered by the circular patterns (c.f., Fig. 3b) formed into the porous section, whereas the uncovered calcite was etched away, forming the microchannel to the upstream and downstream of the porous section (Fig. 2VII). The SEM photos in Fig. 3c,d clearly show that the top surfaces of the posts are smooth, and the edges are precise and clean, demonstrating the precision of the fabrication. Figure 3e shows a further blown-up view of one single pillar, demonstrating the very smooth lateral surfaces of the etched pillar with virtually sub-micrometer roughness. The sample was again rinsed with DI water and then dried with nitrogen. Two holes were drilled at the two ends of the microchannel to serve as the inlet and outlet. Following the drilling, a second microscope slide was adhesively bonded to form a closed micromodel, and two nanoports (IDEX Health & Science, N-333) were attached to the micromodel for fluid delivery^23^ (Fig. 2VIII)), completing the entire fabrication process.

### Experimental procedure

To perform flow measurements with the calcite-based micromodel, an experimental apparatus was constructed around an inverted epi-fluorescence microscope (Olympus, IX71). Details regarding the experimental apparatus and flow test procedures are provided in the Supplementary Information. As a proof-of-concept and a demonstration of the micromodel capability, the preliminary tests presented in this study used 0.01% HCl (or water for micro-PIV test) as the working fluid to ensure a controlled reaction with the calcite. This combination was chosen for its simple dissolution kinetics as dictated by Ref.^45^,

1 $$\begin{aligned} \text {CaCO}_3(\text {s}) + 2\text {HCl}(\text {aq}) \rightarrow \text {CaCl}_{2}(\text {aq}) + \text {CO}_2(\text {aq}) + \text {H}_2\text {O}(\text {l}). \end{aligned}$$

Both fluorescent microscopy and micro-PIV are tested as flow diagnostic tools. As detailed in the Supplementary Information, to perform fluorescent microscopy, the working fluid was tagged with a fluorescence dye, Rhodamine B (RhB, Acros Organics, 98+% pure, CAS:81-88-9), whereas for the micro-PIV measurements, the working fluid was seeded with 1- $\upmu$m polystyrene fluorescent particles (Invitrogen F8819). A new micromodel was used for each experimental run, and the flow rates were kept at 50 $\upmu$l/min and 0.02$\upmu$l/min, corresponding to nominal Reynolds numbers of 7.5 and $3\times 10^{-3}$ based on the average pore diameter, for the fluorescent microscopy and micro-PIV experiments, respectively. In this study, 1000 raw micro-PIV images were processed with a multi-pass approach combined with the correlation averaging scheme^55^ employing the open source MATLAB code, PIVlab^56^. The size of the interrogation windows was 64$\times$64 pixels, with 50% overlap, which yielded a spatial resolution of 84 $\upmu$m and a velocity vector spacing of 42 $\upmu$m.

## Results and discussion  

In this section, we present several representative results that were obtained employing the calcite-based micromodel developed in this work and the imaging techniques described above. Although this paper primarily focuses on the introduction and description of the novel methodology, the results serve as a demonstration of the potential and capability of the method. A more systematic quantification of the physics underlying pore-scale mineral dissolution will be pursued in a separate study.

### Overall dissolution dynamics

**Fig. 4:**
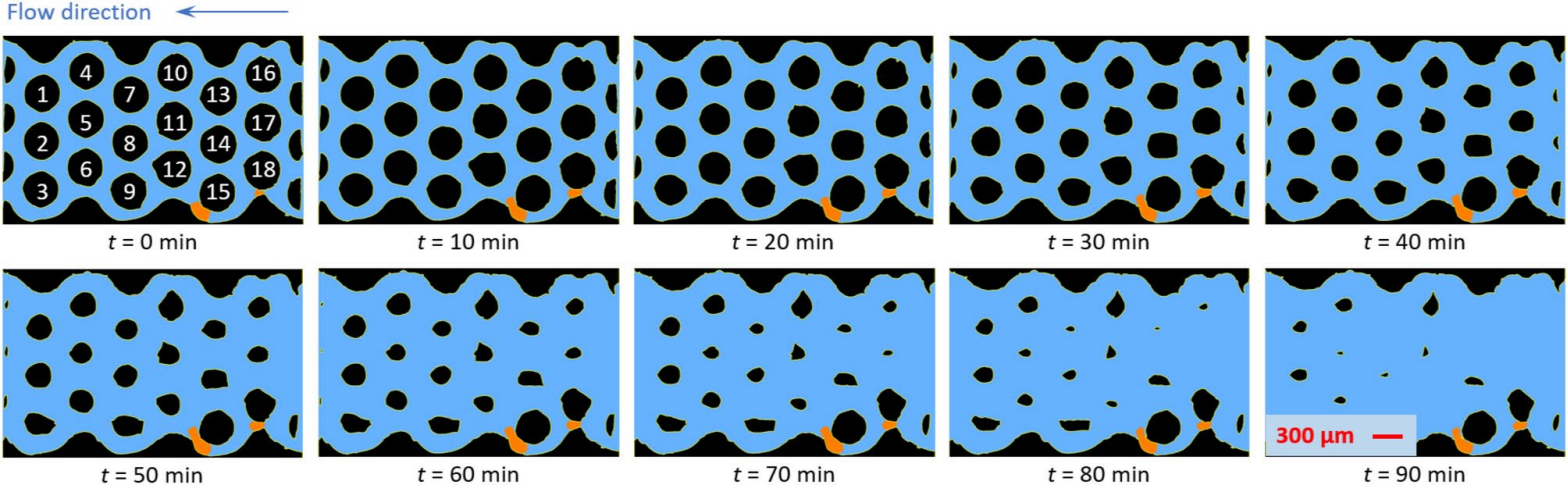
Evolution of the calcite porous section over time subject to dissolution by HCl. Herein, the black, light blue and orange regions indicate calcite, HCl solution, and trapped air bubbles, respectively. The calcite grains in the top left panel are numbered to facilitate easy reference to each individual grain in the discussion.

Figure 4 shows the evolution of the calcite grains in the porous section over time. The bulk flow is from right to left in this experiment and the progressive erosion of the grains is due to mineral dissolution of calcite in the acidic aqueous phase. The two air bubbles were intentionally introduced into the porous section during the pre-saturation of the porous section with HCl and remained trapped in the porous section throughout the entire experimental run. Their presence helps us understand their impact on the dissolution behavior. From Fig. 4, it can be seen that almost all grains in the porous section progressively shrink over time. The erosion, due to dissolution, is slightly faster for the upstream grains compared to the downstream grains. This is to be expected as the HCl solution is at its highest concentration as it enters the porous section, where it yields higher reaction rates. In the downstream portion of porous section, the chemical composition of the working fluid changes due to the dissolution reaction which consumes HCl and produces Ca$^{2+}$ resulting in slower reactions (Eq. (1)). Another interesting observation is that not all the calcite grains maintain their circular symmetry throughout the dissolution process. For instance, grains NO.1 and NO.2 start with a circular shape and gradually evolve into a drop-like shape with a round leading edge and a sharper trailing edge (see $t=80$ min in Fig. 4 for instance). This shape shows a spanwise symmetry and a slight streamwise asymmetry, which suggests that a spatial variability of the reaction kinetics exists at the grain-scale, presumably due to the combined effect of local concentration gradients and local velocity field that warrants further investigation. On the contrary, grains NO.3 and NO.9 only undergo appreciable dissolution on the top surfaces, leading to nearly semi-circle shapes (Fig. 4, $t=80$ min). We believe that this interesting behavior is caused by the two trapped air bubbles upstream of grain NO.9, which significantly obstruct the flow through the bottom regions of the porous section. This prevents new HCl from reaching the bottoms of the grains, where the reaction is evidently reduced, while diverting high-concentration HCl toward the top of grains NO.3 and NO.9, enhancing local erosion. The strongest evidence supporting the flow diversion induced by the bubbles is clearly visible in grains NO.15 and NO.18 whose erosion is remarkably different from what described above. While grain NO.18 displays some degree of erosive activity, NO.15 appears to be barely altered, indicating that the flow diversion around NO.18 is impeding HCl from reaching NO.15 (sheltering effect). These examples clearly demonstrate how the heterogeneity of the flow, in this case induced by the presence of the air bubbles in the form of preferential flow paths (previously revealed by Blois et al.^57^), tend to disrupt the flow periodicity in the original geometry, resulting in significant spatial variability in reactive transport. These observations indicate the need for detailed pore-scale studies, similar to the current one, that challenge previous assumptions of uniform concentration and dissolution rate^16^.

**Fig. 5:**
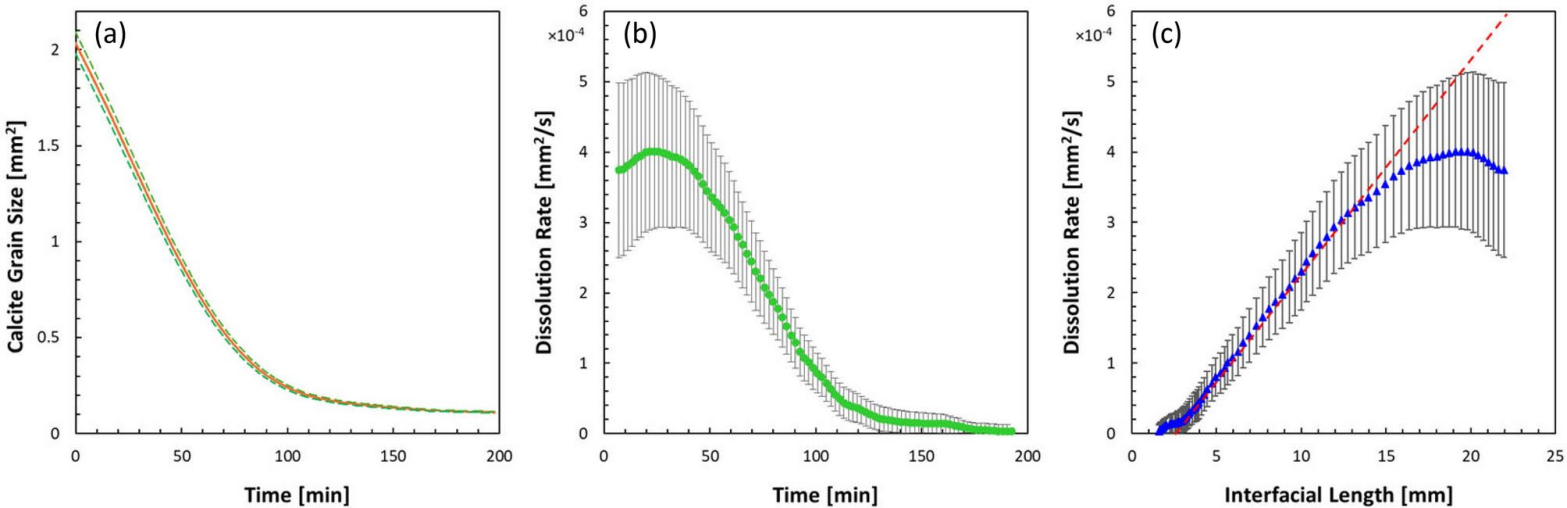
Plots describing the mineral dissolution process of the calcite grains over time: (**a**) time variation of the overall calcite grain area; (**b**) dissolution rate of calcite grains calculated using Eq. (2); (**c**) dissolution rate as a function of interfacial length. In panel (**a**), the green lines indicate the upper and lower bounds of the measurement due to measurement uncertainty, and in panels (**b,c**), the error bars indicate the uncertainties, which were propagated from the data shown in panel (**a**).

Figure 5 shows the progress of mineral dissolution global metrics with time. In this quantification, only the labeled grains shown in Fig. 4 were considered, to minimize the boundary effect. Additionally, the micromodel used in this study is 2D, which means that the dimension in the depth direction is much smaller than the other two dimensions, and the top and bottom of the calcite grains are both sealed with UV glue, limiting reaction only to the lateral walls of the grain. Since the grains are flat with a constant height, as demonstrated by the SEM photos in Fig. 3c,d, variation in the vertical direction is expected to be minimal and negligible. Therefore, the instantaneous volume of the calcite material can be accurately represented by the total area of the calcite grains multiplied by the depth (30 $\upmu$m). Figure 5a shows the evolution of the total area of the calcite grains within the porous section. As expected, the total area (and thus the volume) of the calcite grain decreases monotonically with time following nearly an exponential decay, until the total area eventually approaches zero. Uncertainties in the calcite area calculation originating from a statistical analysis of the image segmentation results are reported in the figure.

Based on the data shown in Fig. 5a, the rate of dissolution $R_\text {exp}$ was calculated using the finite difference scheme following the equation below,

2 $$\begin{aligned} R_\text {exp}=\frac{A_\text {Cal,1}-A_\text {Cal,2}}{t_\text {1}-t_\text {2}}, \end{aligned}$$

where $A_\text {Cal,1}$ and $A_\text {Cal,2}$ are the instantaneous areas of the calcite grains at $t_1$ and $t_2$, respectively. The data was then smoothed using a moving average filter with a window size of 100 data points and plotted in Fig. 5b. Here only every 250th data points are shown to avoid excessive data overlapping. The uncertainties, indicated by the errors bars, were propagated from the uncertainties in the calcite area calculation. As shown in Fig. 5b, the dissolution rate starts at 3.74$\times 10^{-4}$ mm$^2$/s and gradually increases displaying a maximum at 4.0$\times 10^{-4}$ mm$^2$/s which occurs 20 min after the start of flow. The slightly lower initial dissolution rate is unexpected but presumably due to the relatively low flow rate upon the initialization of the experimental run, which typically takes a few to tens minutes to reach steady state, calling for further investigation. After $t=30$ min, the dissolution rate dramatically decreases with time, following a very similar exponential decay as the curve in Fig. 5a. We conjecture that overall, the decay in dissolution rate is primarily due to the shrinking of the calcite grains leading to a substantial reduction in the reaction area around each grain, as further elaborated below.

As mentioned previously, the dissolution kinetic of calcite subject to HCl aqueous solution is dictated by Eq. (1), which is thought to occur via three parallel reactions, as shown below^58,59^:

3 $$\begin{aligned} \text {CaCO}_3 + \text {H}^{+} \leftrightarrow \text {Ca}^{2+} + \text {HCO}_3^{-}, \end{aligned}$$

4 $$\begin{aligned} \text {CaCO}_3 + \text {H}_2\text {CO}_{3}\leftrightarrow \text {Ca}^{2+} + 2\text {HCO}_3^{-}, \end{aligned}$$

5 $$\begin{aligned} \text {CaCO}_3 + \text {H}_{2}\text {O} \leftrightarrow \text {Ca}^{2+} + \text {HCO}_3^{-} + \text {OH}^{-}. \end{aligned}$$

According to the transition state theory^59^, each reaction pathway has its own reaction rate and the overall dissolution rate, $R_\text {th}$, is the summation of the rates of all three parallel reactions:

6 $$\begin{aligned} R_\text {th} = Ak_1a_{\text {H}^+}(1-\frac{IAP_1}{K_{\text {eq,1}}})+Ak_2a_{\text {H}_2\text {CO}_3}(1-\frac{IAP_2}{.}{K_{\text {eq,2}}})+Ak_3(1-\frac{IAP_3}{K_{\text {eq,3}}}) \end{aligned}$$

Here, *A* is the reactive surface area, $k_1$, $k_2$, and $k_3$ are the reaction rate constants for the three parallel reactions, respectively; $a_{\text {H}^+}$ and $a_{\text {H}_2\text {CO}_3}$ are the activities of hydrogen ion and carbonic acid; *IAP* and $K_{\text {eq}}$ are the ion activity products and the reaction equilibrium constants, respectively. As indicated by Eq. (6), the reaction rates depend on several factors: the intrinsic mineral properties, such as the amount of reactive surface area, *A*, and the intrinsic rate constants, *k*; external conditions, such as the concentration of “catalyzing” species including H$^+$ and H$_2$CO$_3$; proximity of the reactions to equilibrium ($IAP/K_\text {eq}$). According to Eqs. (3)–(6) and the well-known Noyes–Whitney equation^60,61^, in a perfectly mixed conditions, where mineral dissolution is purely reaction-rate-limited, with negligible concentration gradient and transport hindrance, the dissolution rate should scale linearly with the reactive surface area. In fact in the current experiment, the high flow rate and low HCl concentration enhance convective transport while limiting reaction, rendering the dissolution process largely reaction-limited. Figure 5c shows the dissolution rate as a function of the interfacial length of the calcite grains. Again, since the depth of the calcite grains is a constant, the interfacial length is effectively a measure of the reactive surface area, *A*. As expected, a majority of the curve follows a straight line, as the theory predicts, indicating a linear relationship between the dissolution rate and the interfacial length. The data shows two exceptions indicating transport-limited reaction at the low- and high-end of reaction rates, which correspond to the end and beginning of the experiment, respectively. We believe that at the beginning of the experiment, the dissolution is transport-limited due to the low supply rate of HCl relative to the available calcite surface area. On the contrary, the end of the experiment is transport-limited presumably due to the low availability of calcite, in part owing to the presence of impurities in calcite or blockage by gas bubbles, relative to the supply rate of HCl.

### Pore-scale grain evolution

A big advantage of the microfluidic approach is that it not only provides a big picture of the overall dissolution rates, but also provides the local dissolution rates at virtually every point of the porous section, which has not been possible in experiments performed with core samples or batch reactors. As pointed out by Dutka et al.^62^, the shape evolution data is a more rigorous test of the validity of pore-scale modeling than average measurements such as effluent concentration, as it requires the correct flux at each point on the sample surface at any moment.

**Fig. 6:**
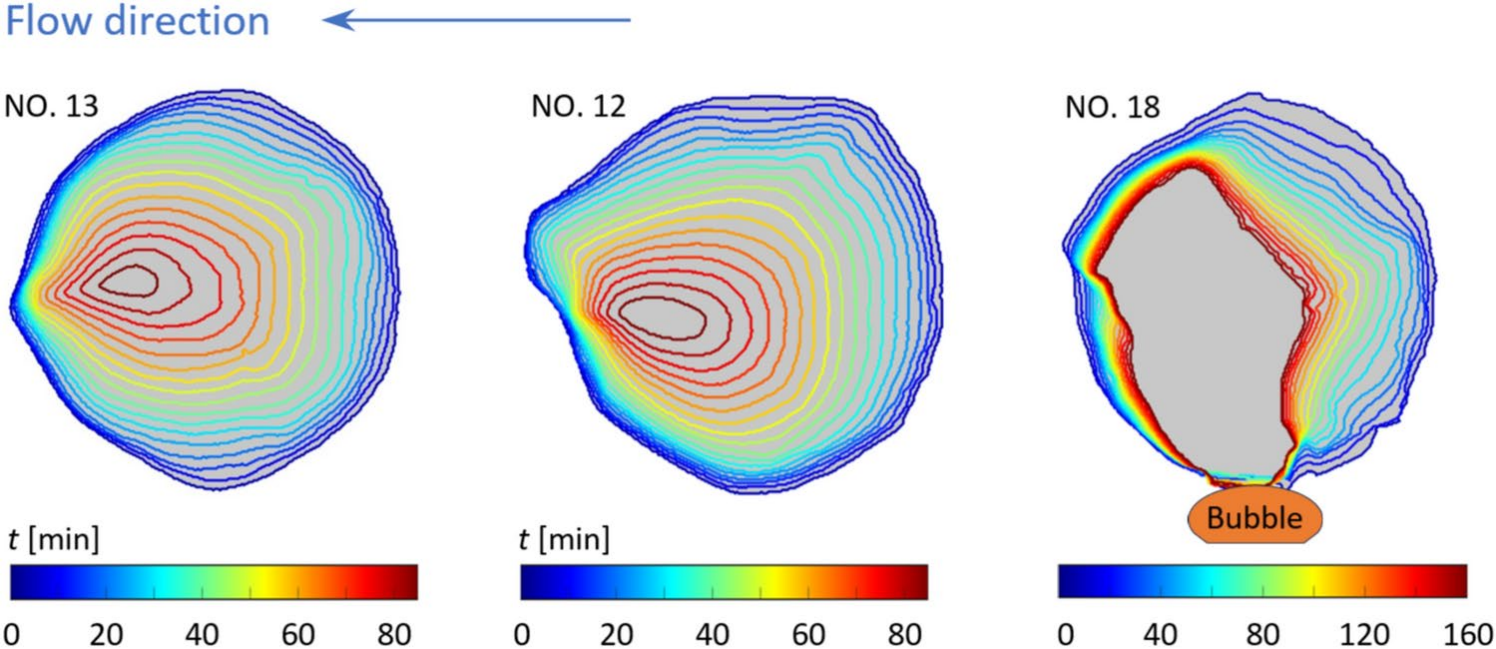
The shape evolution of single grains over time. Herein, grain NO.13, NO.12 and NO.18 are shown as examples. Each contour demarcates the edge of the grain at a given time, which is color coded. Dark blue and dark red indicate the beginning and the end of the process, respectively. Flow direction is from right to left. A gas bubble is observed right below Grain NO.18 as tentatively demarcated herein.

Figure 6 shows the evolution of single grains (i.e., grain NO.13 as labeled in Fig. 4) throughout the dissolution process. As mentioned previously, the calcite post NO.13 remains quite symmetric top to bottom yet with a significant fore-aft asymmetry, with the trailing edge developing a sharp cusp over time, in qualitative agreement with a previous study where a circular gypsum disk was dissolved in water^62^. In this contour plot, as the period of time is evenly spaced between two adjacent contours, the distance between contours measures the local rate of dissolution at the specific time and location. With that in mind, it can be seen that dissolution rate is relatively high at the leading edge of the calcite post, gradually decreases as traveling around the post and reaches a minimum at the middle of the trailing edge. While the local dissolution rate is the driving force that continuously shapes the calcite post, it is in turn governed by the local advective and diffusive transport. As shown by Dutka et al.^62^ in the simulation of a gypsum disk being dissolved in a water flow, the concentration boundary layer thickness is the thinnest at the leading edge and grows substantially as the flow travels around the post (see their Fig. 7 for instance), which introduces the lowest and highest transport resistances at the leading and trailing edges, respectively.

While Fig. 6 [left] presents a nearly symmetric dissolution pattern, presumably produced by a symmetric laminar flow, asymmetric erosion patterns are indicative of asymmetric flows. Figure 6 also shows the contour plots of Grain NO.12 [middle] and NO.18 [right]. Grain NO.12 displays substantial asymmetry between top and bottom, with much higher dissolution rate observed on the top. This behavior is attributed to the asymmetry in the flow around Grain NO.12, as mentioned previously. Due to the presence of the two gas bubbles next to Grain NO.15 and NO.18, the acid flow and thus advective transport through the bottom channel of the porous section is significantly reduced compared to the top side of the grain, causing the grain to be skewed. Similarly, Grain NO.18 undergoes appreciable amount of dissolution at the top, but only minimal dissolution at the bottom. This is because the channel below Grain NO.18 is obstructed by a gas bubble, which shields the grain surface from the acid flow, significantly hindering dissolution therein. As shown in Fig. 6 [right], the grain edge at the bottom hardly moved throughout the entire dissolution process of 160 min long.

### Pore-scale flow field

**Fig. 7:**
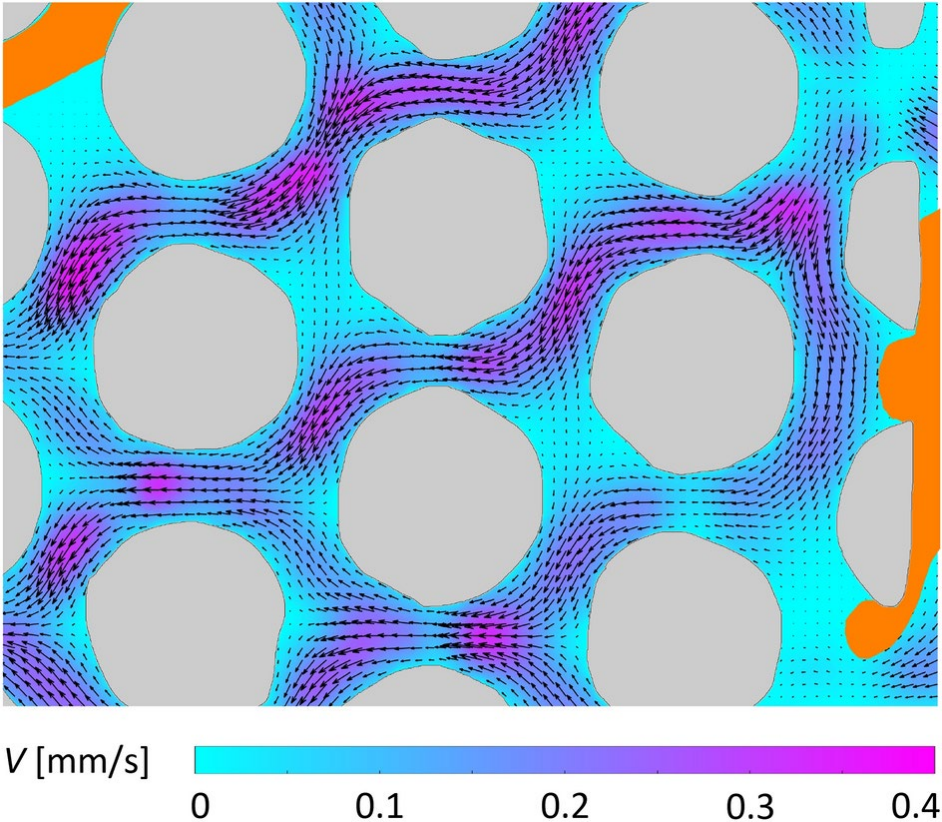
A sample velocity field of water and air through the calcite micromodel at a flow rate of 0.02 $\upmu$l/min ($Re=3\times 10^{-3}$) obtained with micro-PIV. Colored contours and arrows indicate the velocity magnitude and direction, respectively, in the aqueous phase; and gray and orange regions represent solid grains and gas bubbles, respectively.

The innovative design of this present calcite-based micromodel has allowed us to use the advanced micro-PIV technique as a flow diagnostic tool to achieve flow field quantification. As a demonstration, Fig. 7 presents a sample velocity field of water and air through the calcite micromodel at a flow rate of 0.02 $\upmu$l/min. This measurement was performed in a new separate micromodel, where gas bubbles were again manually introduced after the micromodel was saturated with water and the bulk flow of water was introduced from right to left. As expected, high-momentum flows are observed in the narrow regions (i.e., called throats) of the porous section, whereas much lower speed is observed in the open pore spaces due to continuity, in agreement with previous studies^22,24^. In a regularly arranged geometry like this, a single-phase flow would be expected to be symmetric and periodic with streams going right to left meandering around the circular calcite cylinders as shown in our previous work^38^. However, herein the flow is significantly modified due to the presence of a gaseous phase, in the form of trapped air bubbles introduced during the experiment, or CO$_2$ bubbles generated in-situ as a result of chemical reaction between the liquid and solid phases. The gas bubbles substantially block the flow pathways, causing little to no flow in close proximity, highlighting the hindrance effect of the bubbles on advective transport.

It is worth noting that the velocity fields generated with this approach can also enable computations of various scalar fields, such as Péclet (*Pe*) and Damköhler number (*Da*) fields, all involved in the mechanisms controlling the reactive transport and used as characterization metrics of the relative importance of reaction, diffusion, and advection. The Péclet number (*Pe*) and the Damköhler number are (*Da*) defined as^63,64^:

7 $$\begin{aligned} Pe = Vl/D, \end{aligned}$$

8 $$\begin{aligned} Da=kl/V. \end{aligned}$$

Here, *V* is the fluid velocity, *l* is the characteristic length scale (e.g., pore diameter), *D* is the diffusion coefficient, and *k* is the reaction rate constant. Physically, *Pe* defines the ratio of advective to diffusive transport rates, and *Da* defines the ratio of the overall chemical reaction rate to the advective mass transport rate. *Pe* and *Da* fields will provide valuable insights into the fundamental pore-scale mechanisms of mineral dissolution and will be investigated systematically in our future work.

### Outlook and future potential

**Fig. 8:**
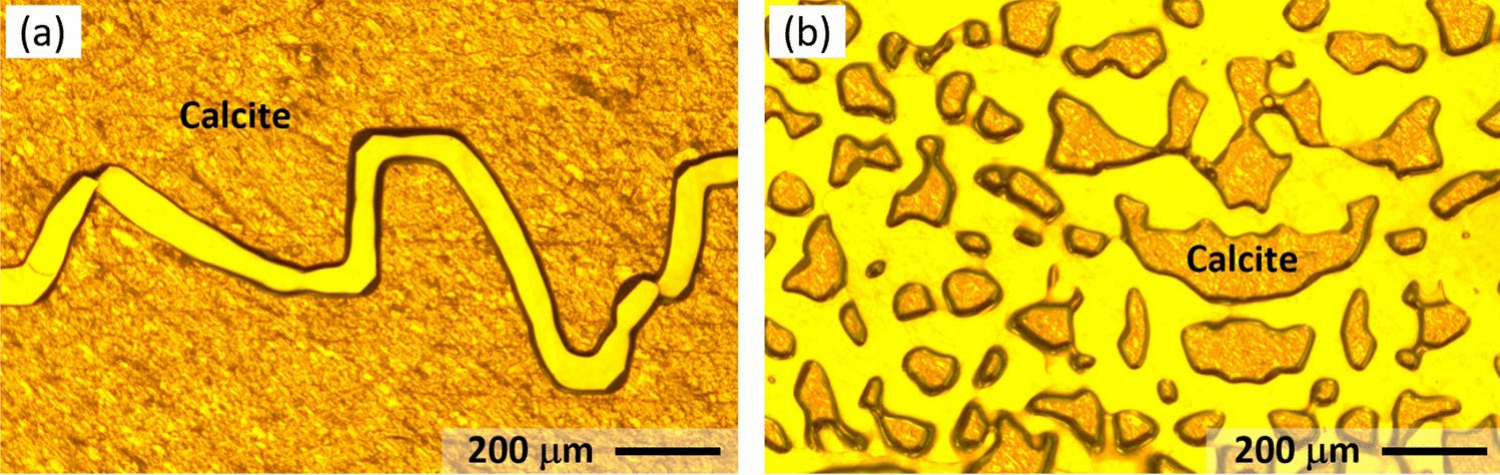
Photos of calcite-based micromodels fabricated with the current approach to represent a naturally occurring fracture (**a**)^15^, and the geology of a heterogeneous reservoir (**b**)^22^. The smallest feature achieved in this batch is $\sim \,30\upmu$m.

The featuring contribution of this work is the introduction of a microfluidic approach that includes 2D reactive porous media and advanced pore flow diagnostics for the study of pore-scale dissolution in porous media. In this pilot study, several simplifications were intentionally adopted. For instance, an idealized pore structure (i.e., regularly arrange circles), uniform wettability, and one single mineral (i.e., calcite) were used, which has helped us to focus on the demonstration and validation of the approach. However, in addition to this basic configuration, it is worth noting the vast possibilities and potentials that this approach has enabled. First, the porous structures can be formed to represent nearly any pattern and geological features as shown in Fig. 8 at no extra cost. Second, the novel material used in the fabrication enables easy and controlled alteration of surface wettability, which can further incorporate spatially heterogeneous wettability via selective UV exposure^54^. Finally, the mineralogy of the micromodel can be altered and diversified via physical and chemical vapor deposition (P/CVD), a widely used materials processing technology in which thin films of common minerals such as metal and silica can be selectively formed. Although microfabrication as a general technology has been used in a couple of recent studies^51,52^, we believe the protocol presented herein is new with better capability, accuracy and potential.

## Summary

A novel methodology has been presented to fabricate 2D calcite-based porous micromodels by combining traditional techniques with innovative microfabrication techniques such as photolithograpy, wet etching and adhesive bonding. To the best of our knowledge, this is the first time that photolithograpy is directly applied to calcite samples, which turned out to work satisfactorily well. Compared with previous designs, this calcite micromodel geochemically and structurally represents real carbonate reservoirs, offers precise control over the structures and chemical properties, and facilitates unobstructed and unaberrated optical access to the pore flow. Additionally, by creatively molding the microchannel with UV glue, we have been able to eliminate unwanted reactions outside the porous section, thus achieving more controlled flow and reactions in the micromodel.

Preliminary pore-scale flow test has been performed in sample micromodels employing advanced flow diagnostics such as epi-fluorescent microscopy and micro-PIV. Average mineral dissolution dynamics has been quantified by direct measurements of dissolution patterns, overall dissolution rate and reactive area. Pore-scale grain evolution has been quantitatively imaged with unprecedented details thanks to the use of microscopy. The preliminary results have revealed the crucial roles of reactive transport and local concentration gradients in mineral dissolution in porous media and call for reconsideration of many assumptions used in traditional and state-of-the-art modeling tools. Additionally, the study has successfully demonstrated the use of micro-PIV to quantitatively investigate the flow driving the chemical reaction in porous media, which will potentially open the door to a series of innovative empirical studies and analysis approaches that were not possible previously. We believe the novel methodology not only offers a novel perspective on the flow dynamics during multiphase mineral dissolution but also paves the way for renewed fundamental understanding and modeling tools with better accuracy.

## Supplementary Information

Supplementary Information.

## Data Availability

The datasets generated during and/or analysed during the current study are available from the corresponding author on reasonable request.
