# Supplementary material for: A novel microfluidic approach to quantify pore-scale mineral dissolution in porous media
Source: Sci Rep. 2025 Feb 21;15:6342. doi: 10.1038/s41598-025-90429-x (PMC11845696; doi:10.1038/s41598-025-90429-x)
Supplement: Supplementary file 1 — Supplementary Information. [file 41598_2025_90429_MOESM1_ESM.pdf]

Supplementary Information: A Novel Microfluidic Approach to Quantify Pore-Scale Mineral Dissolution in Porous Media

Rafid Musabbir Rahman[1], Elliott Niemur[1], Gianluca Blois[2], Farzan Kazemifar[3], Myeongsub Kim[4], and Yaofa Li[1,5,*]

[1]Montana State University, Department of Mechanical & Industrial Engineering, Bozeman, MT 59717, USA.
[2]University of Idaho, Department of Mechanical Engineering, Boise, ID 83702, USA.
[3]San Jose State University, Department of Mechanical Engineering, San Jose, CA 95192, USA.
[4]Florida Atlantic University, Department of Ocean and Mechanical Engineering, Boca Raton, FL 33431, USA.
[5]University of California, Riverside, Department of Mechanical Engineering, Riverside, CA 92521, USA.
[*]yaofa.li@ucr.edu

ABSTRACT

Mineral dissolution in porous media coupled with single- and/or multi-phase flows is pervasive in natural and engineering systems. Dissolution modifies the physical, hydrological, and geochemical properties of the solid matrix, resulting in a complex coupling between local dissolution rate and pore-scale flow. The work reports an innovative microfluidic approach that includes novel 2D reactive porous media and advanced pore flow diagnostics for the study of pore-scale dissolution in porous media with unprecedented details. The 2D microfluidic porous media, called micromodels, were fabricated in calcite by combining photolithography and wet etching, which not only offers precise control over the structural and chemical properties, but also facilitate unobstructed optical access to the pore flow, significantly improving over existing methods. We believe the work represents the first of its kind as it for the first time directly applies photolithography to calcite samples and demonstrates the use of particle image velocimetry to investigate chemical reactions in porous media. The preliminary results have revealed the crucial roles of local concentration gradients in mineral dissolution and call for reconsideration of many assumptions used in the current modeling tools, which paves the way for renewed fundamental understanding of reactive transport and improved modeling tools with better accuracy.

1 Micromodel Fabrication

The micromodel fabrication procedures are illustrated in detail in Fig. S1 to complement Fig.2 in the manuscript. While only 8 major steps were included in Fig. 2, this detailed protocol consists of 15 steps, including all necessary operations to reproduce the micromodel as listed below. As the starting point, a bulk calcite crystal (VWR, Iceland Spar, 470025-522) of $\sim 30 \times 20 \times 5\,\mathrm{mm}^3$ was purchased and used as received.

1. The calcite crystal was glued to a glass slide using UV glue to facilitate polishing in the next step. The sample was cured with a UV lamp for 30 minutes while securely clamped.
2. The top surface of the calcite was ground and polished using a grinder (Allied High Tech 5-2600) combined with diamond discs (Allied High Tech 50-80800) and the alcohol-based diamond slurries (Allied High Tech 90-3AB6-G).
3. The polished calcite surface was cleaned with acetone and attached to a second glass slide (Ted Pella, Inc. Product NO:260600) using the UV glue (Thorlabs, NOA81), which was cured with a UV lamp for 30 minutes while securely clamped. Then the clamp was removed and the sample was cured for another 30 minutes under the UV light to ensure secure bonding between the calcite layer and the glass slide.
4. The entire assembly was sliced in the middle of the calcite using an automatic petrographic thin section machine (Pelcon Automatic Thin Section Machine) to separate the two glass slides. While the part with the polished calcite was kept for the next step, the part with the unpolished side was disposed of.
5. The sliced surface (now at the top) of the attached calcite was ground using silicon carbide papers. The grinding process started with a silicon carbide paper with a grit size of 400 and gradually refined to the grit size 800 to ensure efficiency as well as grinding quality. The targeted thickness of the calcite in this step is 100-120 μm. Specifically, the process

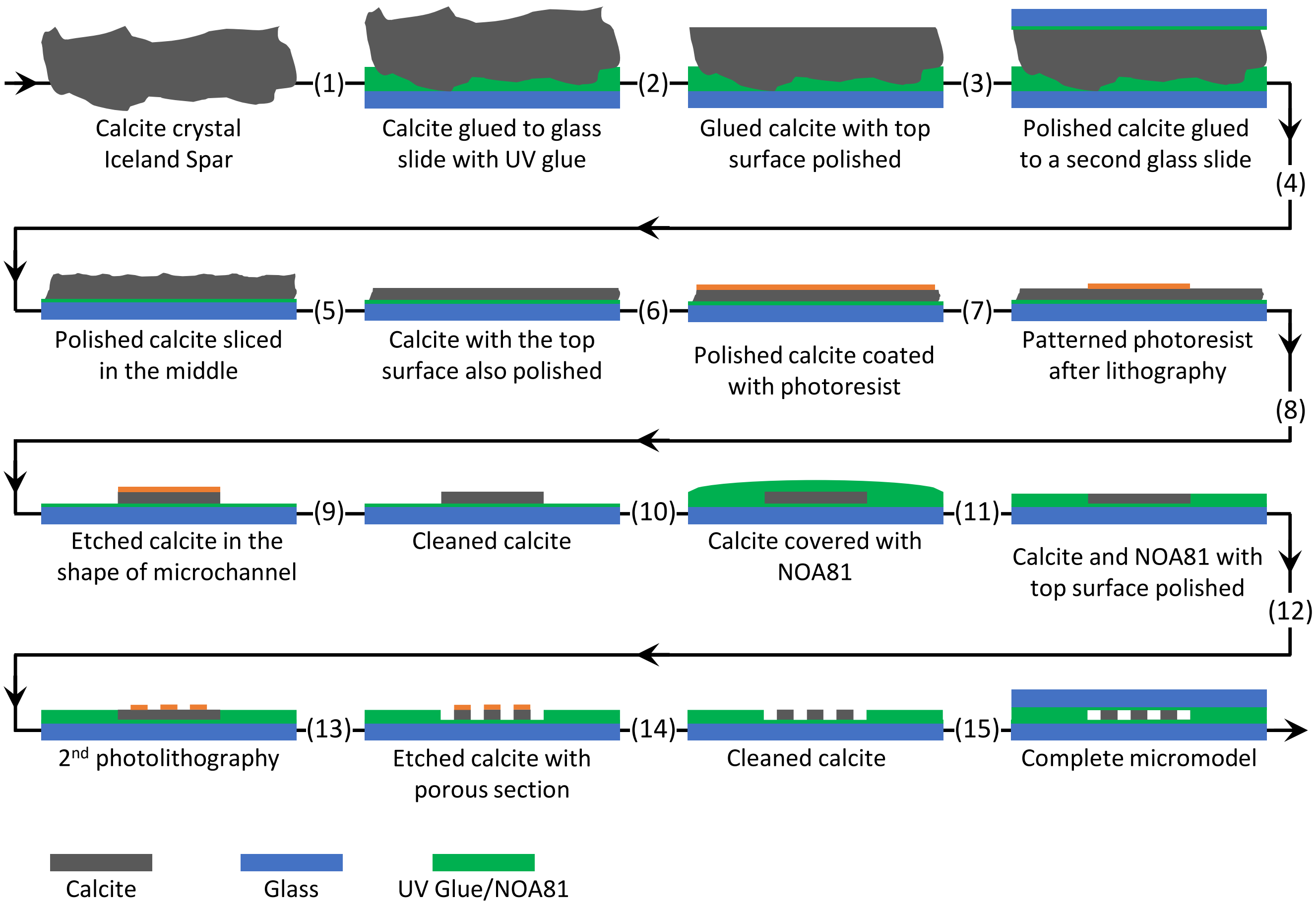

Figure S1. Schematic diagram of the experimental setup. The apparatus is constructed around an inverted epi-fluorescence microscope (Olympus, IX71), which is equipped with a green LED (Thorlabs SOLIS-525C) and a CMOS camera (Andor, Neo 5.5 sCMOS). The camera and LED are both controlled and synchronized by a function generator and a PC. The micromodel is mounted on the translation stage of the microscope, the flow through which is controlled by a syringe pump (Harvard Apparatus, PHD 2000).

started with a 400 grit paper for 3 minutes and 45 seconds, following which a 600 grit paper was used for 1 minute and the process was completed with a 800 grit paper for 5 minutes resulting in a calcite thickness of approximately 118 μm as measured by a digimatic micrometer (Mitutoyo, 293-821-30). These polishing and grinding processes were again done on the Allied High Tech Metprep 3™ grinder/polisher with a power head (Allied High Tech 5-2600).

6. At this point, a uniform layer of calcite of approximately 118 μm was successfully formed on the glass slide, which was then ready for the first photolithography process. To perform photolithography, a photomask (Mask I) that carries the shape of the main channel was used. The photomask was designed in Adobe Illustrator® and printed by a third-party company (CAD/Art Services, Inc.). A 9 μm thick layer of photoresist (MicroChemicals, AZ®10XT) was spin-coated on the calcite sample at a spinning speed of 2000 rpm for 45 s. Then the sample was soft baked for 6 minutes at 115 °C, following which the sample was allowed to rest for 1 hour to thermally equilibrate with the environment.

7. The photoresist was subsequently exposed under UV light at 3.36 mW/cm^2 for 106 s, and developed in a photoresist developer (MicroChemicals, AZ®300 MIF) for 15 minutes. Finally, the sample was rinsed with DI water and dried with high-purity nitrogen stream to complete the photolithography process.

8. Then the calcite layer was wet etched in 2% hydrochloric acid (HCl) for 4 minutes or until the uncovered portion was all etched through.

9. The sample was rinsed with acetone, methanol and DI water and then dried with nitrogen. These procedures produce a thick calcite layer of 118 μm that is adhesively bonded to the glass slide and shaped as the microchannel.

10. A layer of UV glue was dispensed over the dumbbell-shaped calcite to cover the glass slide. The glue was degassed under vacuum for 30 minutes to remove any bubbles from the glue, following which the glue was cured for 10 minutes under UV light (Thorlabs, SOLIS-365C), resulting in an approximately 0.5 mm thick UV glue layer covering the calcite.

11. The cured glue along with the covered calcite was then ground using the same Allied High Tech Metprep 3^{TM} grinder/polisher with the power head (Allied High Tech 5-2600) to reach a thickness of about 30 μm. For this step, silicon carbide paper of 600 grit size was used for 6 minutes and 50 seconds, eventually exposing the dumbbell-shaped calcite at the top surface, which is surrounded by the cured UV glue.

12. The polished sample was then taken to perform the second photolithography process with Mask II to form the porous structures on the calcite. The materials and protocols are largely the same as the first photolithograpy process with only one difference that the UV exposure of the photoresist was done with the aid of a customized mask aligner, to ensure the porous pattern is well-aligned with the calcite. Following a sequential processes of UV exposing, developing, and cleaning, the sample was ready for the second etching process.

13. The sample was etched again by immersing it in 0.5% HCl solution for 10 minutes. After etching, the regions that were covered by the circular patterns formed into the porous section, whereas the uncovered calcite was etched away, forming the microchannel to the upstream and downstream of the porous section.

14. The sample was again rinsed with DI water and then dried with nitrogen. Two holes were drilled at the two ends of the microchannel to serve as the inlet and outlet.

15. Following the drilling, a second microscope slide was adhesively bonded to form a closed micromodel.

    Finally, two nanoports (IDEX Health & Science, N-333) were attached to the micromodel for fluid delivery, completing the entire fabrication process.

Experimental Procedure

Apparatus and working fluid

To perform flow measurements with the calcite-based micromodel, an experimental apparatus was constructed around an inverted epi-fluorescence microscope (Olympus, IX71) as shown in Fig. S2. The micromodel was mounted on an automated translation stage, with the inlet and outlet connected to a syringe pump (Harvard Apparatus, PHD2000) and a glass beaker serving as a waster container, respectively. The microscope was equipped with a green LED (Thorlabs, SOLIS-525C) and a scientific CMOS camera (Andor, Neo 5.5 sCMOS), which are in turn synchronized and controlled by a function generator (Berkeley Nucleonics, 565-4C) and a PC. The LED and the camera were used to illuminate and image the flow, respectively.

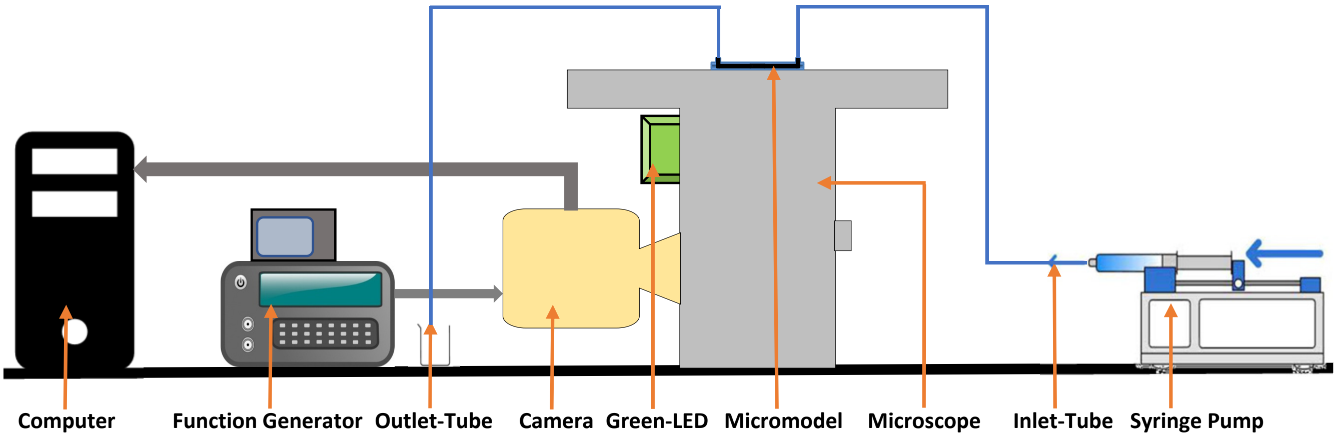

Figure S2. Schematic diagram of the experimental setup. The apparatus is constructed around an inverted epi-fluorescence microscope (Olympus, IX71), which is equipped with a green LED (Thorlabs SOLIS-525C) and a CMOS camera (Andor, Neo 5.5 sCMOS). The camera and LED are both controlled and synchronized by a function generator and a PC. The micromodel is mounted on the translation stage of the microscope, the flow through which is controlled by a syringe pump (Harvard Apparatus, PHD 2000).

As a proof-of-concept and a demonstration of the micromodel capability, the preliminary tests presented in this study used 0.01% HCl (or water for micro-PIV test) as the working fluid to ensure a controlled reaction with the calcite. This combination was chosen for its simple dissolution kinetics as dictated by[1],

$$CaCO_3(s) + 2HCl(aq) \rightarrow CaCl_2(aq) + CO_2(aq) + H_2O(l) \tag{1}$$

As detailed below, to perform fluorescent microscopy, the working fluid was tagged with a fluorescence dye, Rhodamine B (RhB, Acros Organics, 98+% pure, CAS:81-88-9), whereas for the micro-PIV measurements, the working fluid was seeded with 1-μm polystyrene fluorescent particles (Invitrogen F8819). For the fluorescent microscopy, a 0.5% HCl stock solution was prepared by diluting a 37% HCl solution with DI water, whereas a 10 mM RhB stock solution was prepared by dissolving 47.9 mg RhB powder in 100 ml DI water. Then 0.5 ml RhB stock solution, 0.16 ml 0.5% HCl solution, and 7.34 ml DI water were mixed, resulting in the final HCl and RhB concentrations of 0.01% and 0.625 mM, respectively. Similarly, for the micro-PIV, 10 μl stock solution containing particles of 1 μm in diameter (2% v/v solid), 0.165 ml of 0.5% HCl stock solution, and 7.835 ml of DI water were mixed, resulting in the final HCl concentration of 0.01%, and a particle volume fraction of 2.5×10^{-5}. The polystyrene particles have a density of 1.05 g/ml, which closely matches that of water, ensuring that the particles are "neutrally buoyant" and thus faithfully follow the flow. The volume fraction of solid particles was kept low to avoid significant alterations in the properties of the aqueous phase. A new micromodel was used for each experimental run, and the flow rates were kept at 50 μl/min and 0.02μl/min, corresponding to nominal Reynolds numbers of 7.5 and 3×10^{-3} based on the average pore diameter, for the fluorescent microscopy and micro-PIV experiments, respectively.

Fluorescent microscopy

In the fluorescent microscopy measurement, the LED light source (Thorlabs SOLIS-525C) with a nominal wavelength of 525 nm filtered by a bandpass filter (HQ 535/50) was used to illuminate the sample. The emitted fluorescence from the flow was passed through another bandpass filter (HQ 610/75), focused by a 4x microscope objective (Olympus PLN 4x) to the camera (Andor Neo 5.5). As shown in Fig. S3a, the field of view (FOV) created with this configuration is 4.16×1.95 mm^2, and the solid grain and aqueous phase appear as the dark and bright regions, respectively. Grayscale images were captured at 2 frames per second (fps), which was sufficient to temporally resolve the dynamic evolution of the geometry of the solid matrix[2]. The acquired gray-scale images were then analyzed and segmented using an in-house MATLAB code based on the intensity of each pixel[3]. The instantaneous spatial distribution and area of the grains were calculated by counting the number of pixels occupied by the white regions as shown in Fig. S3b. Additionally, once the solid and liquid phases were identified, the interfaces were determined by finding the edges of the solid phase, further producing quantification of the interfacial area for reaction. It is worth noting that this single-color microscopy is only able to differentiate the aqueous phase which is tagged with the fluorescent dye (bright regions herein), whereas all other phases including the solid grains and any gaseous phase, potentially trapped and/or produced by the reaction, all appear as dark regions as shown in Fig. S3a. However, in this particular experiment, the gas phase shows up slightly brighter than the background grains, presumably due to the presence of the gas-liquid interfaces that help scatter fluorescent light, allowing us to manually separate the gas phase from the grains. This manual processing step was possible due to the fact that the trapped gas bubbles do not substantially change over time, making this task feasible with a reasonable amount of manual effort. Although the present optical setup was sufficient to perform the current experiments, where reaction-produced CO_2 was instantaneously dissolved in the aqueous phase, more rigorous quantification of a three-phase flow with the CO_2 evolving as a separate phase will necessitate the use of dual-color fluorescent microscopy as demonstrated in our previous work[4,5].

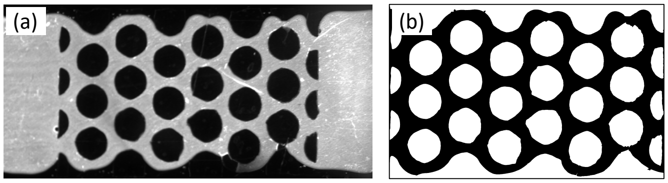

Figure S3. (a) A raw gray-scale fluorescent image with the aqueous phase and the grains shown as bright and dark regions, respectively; (b) a segmented image with the aqueous phase and the grains shown as black and white regions, respectively. The segmented image was cropped to show the region of interest only (ROI). We note that in this particular micromodel only four rows of micropillars were formed in the porous section, as opposed to the five rows in the original design. This was caused by slightly excessive etching in step IV, leading to a microchannel that is slightly narrower than the designed width of 2 mm. This issue can be mitigated and eliminated by making the designed channel width slightly greater than the porous section width.

Particle image velocimetry

In micro-PIV, the experimental setup and procedures are largely similar to that used in the fluorescence microscopy with three major modifications: 1) instead of fluorescent dye, the aqueous phase was seeded with 1 μm neutrally buoyant fluorescent particles as flow tracers (ThermoFishere F8823); 2) a 10x objective was used for better spatial resolution, producing a smaller FOV ($1.66 \times 1.40\,\mathrm{mm}^2$); 3) the camera was run at a higher frame rate (20 fps) to temporally resolve the flow. Two consecutive images of the particles are recorded with a prescribed time delay, $\Delta t = 50\,\mathrm{ms}$. The LED was pulsed by the function generator to produce an effective exposure time of 5 ms per frame, which is critical to avoid any streaks of the tracer particles as they travel with the flow. To generate velocity vector fields from the raw images of particles, image masking was first applied to each image to identify the locations of the solid grains and trapped gas bubbles, where tracer particles were not present. Then each image was subdivided into small areas, called interrogation windows. The average displacement of the tracer particles $\Delta\mathbf{x}$ within each interrogation window was determined statistically from cross-correlation analysis of consecutive images. The velocity vector $\mathbf{u}$ for each interrogation window was then obtained by dividing the displacement $\Delta\mathbf{x}$ by the prescribed Δt[6]. In this study, 1000 raw micro-PIV images were processed with a multi-pass approach combined with the correlation averaging scheme[7] employing the open source MATLAB code, PIVlab[8]. The size of the interrogation windows was 64×64 pixels, with 50% overlap, which yielded a spatial resolution of 84 μm and a velocity vector spacing of 42 μm.

References

1. Song, W., de Haas, T. W., Fadaei, H. & Sinton, D. Chip-off-the-old-rock: the study of reservoir-relevant geological processes with real-rock micromodels. *Lab on a Chip* **14**, 4382–4390 (2014).

2. Singh, R. *et al.* Real rock-microfluidic flow cell: A test bed for real-time in situ analysis of flow, transport, and reaction in a subsurface reactive transport environment. *J. contaminant hydrology* **204**, 28–39 (2017).

3. Li, Y., Blois, G., Kazemifar, F. & Christensen, K. T. High-speed quantification of pore-scale multiphase flow of water and supercritical co2 in 2-d heterogeneous porous micromodels: Flow regimes and interface dynamics. *Water Resour. Res.* **55**, 3758–3779 (2019).

4. Blois, G., Barros, J. M. & Christensen, K. T. A microscopic particle image velocimetry method for studying the dynamics of immiscible liquid–liquid interactions in a porous micromodel. *Microfluid. Nanofluidics* **18**, 1391–1406 (2015).

5. Kazemifar, F., Blois, G., Kyritsis, D. C. & Christensen, K. T. A methodology for velocity field measurement in multiphase high-pressure flow of co2 and water in micromodels. *Water Resour. Res.* **51**, 3017–3029 (2015).

6. Santiago, J. G., Wereley, S. T., Meinhart, C. D., Beebe, D. J. & Adrian, R. J. A Particle Image Velocimetry System for Microfluidics. *Exp. Fluids* **25**, 316–319, DOI: 10.1007/s003480050235 (1998).

7. Meinhart, C. D., Wereley, S. T. & Santiago, J. G. A piv algorithm for estimating time-averaged velocity fields. *J. Fluids Eng.* **122**, 285–289 (2000).

8. Thielicke, W. & Sonntag, R. Particle image velocimetry for matlab: Accuracy and enhanced algorithms in pivlab. *J. Open Res. Softw.* **9** (2021).
